# Renal thromboembolism during treatment with recombinant activated factor VII (rFVIIa) in a child with hemophilia B with factor IX inhibitors

**DOI:** 10.1186/s12887-014-0315-2

**Published:** 2014-12-17

**Authors:** Danko Milošević, Ernest Bilić, Danica Batinić, Mirjana Poropat, Ranka Štern-Padovan, Slobodan Galić, Daniel Turudić

**Affiliations:** Department of Pediatrics, Division of Nephrology, Clinical Medical Centre Zagreb, University of Zagreb School of Medicine, Kišpatićeva 12, 10000 Zagreb, Croatia; Department of Pediatrics, Division of Hematology and Oncology, Clinical Medical Centre Zagreb, University of Zagreb School of Medicine School of Medicine, Kišpatićeva 12, 10000 Zagreb, Croatia; Department of Nuclear Medicine and Radiation Protection, Clinical Medical Centre Zagreb, University of Zagreb School of Medicine, Kišpatićeva 12, Zagreb, 10000 Croatia; Department for Diagnostic and Interventional Radiology, Clinical Medical Centre Zagreb, University of Zagreb School of Medicine, Kišpatićeva 12, Zagreb, 10000 Croatia; University of Zagreb School of Medicine, Šalata 3, Zagreb, 10000 Croatia

## Abstract

**Background:**

Serious thromboembolic events connected with rFVIIa therapy in hemophilia patients are rare. Only three cases are reported in children, all of them with hemophilia A.

**Case presentation:**

We present unique case of patient with hemophilia B and high titer inhibitors to coagulation FIX, who developed severe renal damage due to thromboembolic event during rFVIIa therapy, associated with unsuspected renovascular anomalies.

**Conclusion:**

Caution is necessary if hematuria B requires administration of rFVIIa. US color doppler renal imaging before and after drug administration should be sufficient as an early warning.

## Background

Recombinant factor VIIa (rFVIIa, Novoseven®) has been shown to induce hemostasis in hemophilia patients with inhibitors against factor VIII or factor IX independent of factor VIII/factor IX. rFVIIa initiates hemostasis by forming a complex with tissue factor (TF) exposed as a result of vessel wall injury. Pharmacologic doses of rFVIIa can enhance thrombin generation. Recombinant activated factor VII (rFVIIa, Novoseven®) is in regular clinical use in haemophilia B patients with high-titer inhibitors to coagulation factor IX (FIX) since 1996 [1]. Potential adverse event of rFVIIa therapy is pathological blood clotting. However, when rFVIIa is used in labeled indications such adverse event is rare and did not appear to be dose-related. The incidence of serious thromboembolic events after treatment with rFVIIa in hemophilia patients with inhibitors appears to be much less than 1%, with only three cases reported in children, all of them with hemophilia A and predisposing factors [2-4].

We present the patient with hemophilia B and high titer inhibitors to coagulation FIX who was treated with rFVIIa for severe life-threatening hematuria. Although hematuria was successfully treated, renal thromboembolic adverse event associated with unsuspected vascular anomalies resulted in severe renal damage. To our knowledge, this is the first case of tromboembolic event connected with rFVIIa therapy with this set of symptoms presented exclusively on kidney with underlying vascular anomalies. The second normal kidney was fully spared.

## Case presentation

**Figure 1:**
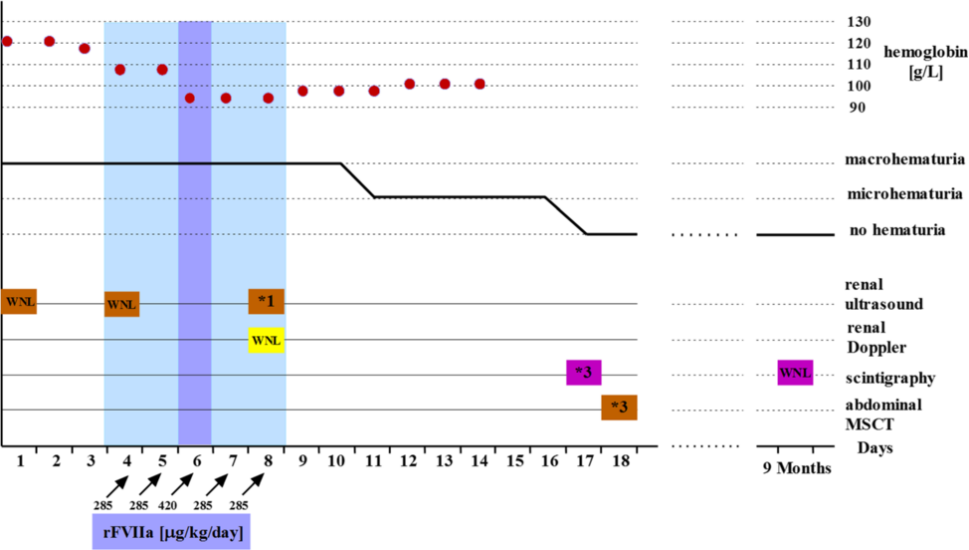
**Clinical, diagnostic and medication follow up.** Blue shaded areas indicate days of activated recombinant factor VII (rFVIIa) therapy. Orange boxes indicate times of renal US. WNL - within normal limits, *1 - left pyelon dilation. Inhomogenous content compatible with pyelon clotting. Yellow box indicate time of renal Doppler, WNL - within normal limits. Purple boxes indicate times of renal scintigraphy, *2 – functional abnormality of the left kidney, WNL - within normal limits. Brown box indicates time of abdominal MSCT, *3 – renovascular anomalies of left kidney.

**Figure 2:**
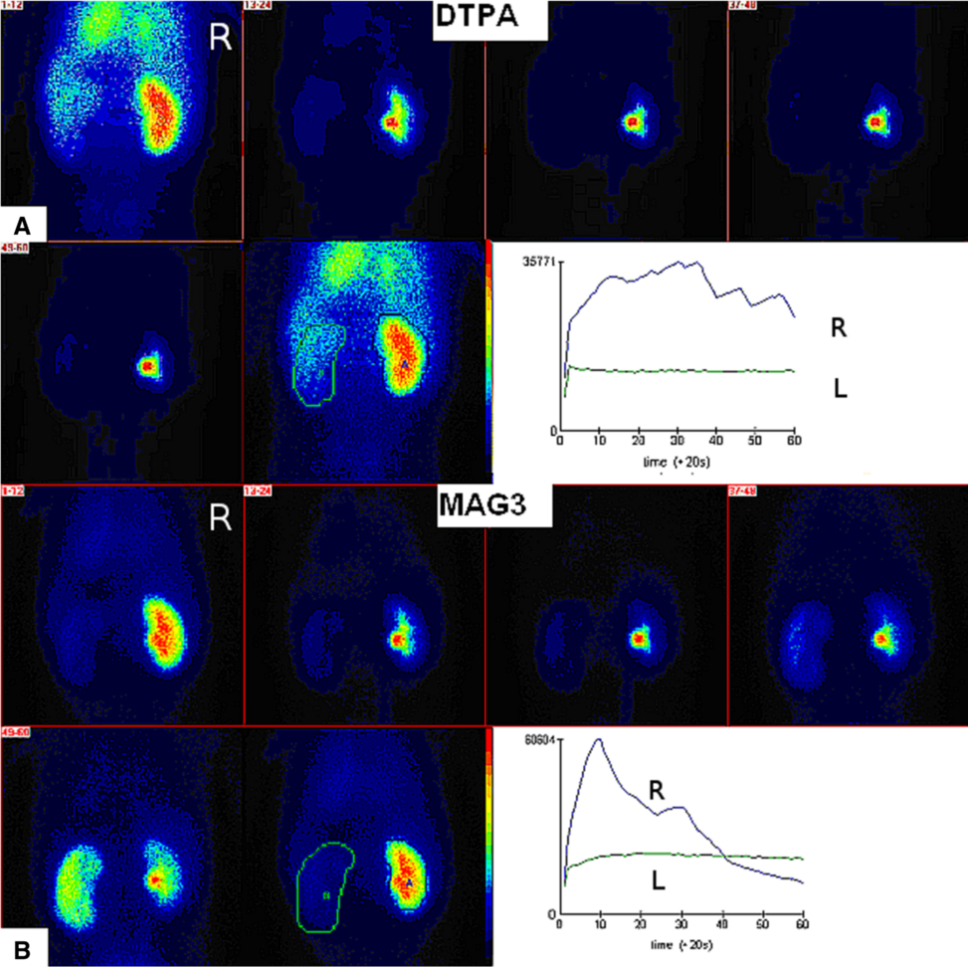
**Renal imaging in posteroanterior position (R = right kidney, L = left kidney). A**. 99mTc-DTPA renal scintigraphy shows vascular bed over the left kidney without visualization of the parenchyma with practically afunctional renographic curve of the same kidney. **B**. 99mTc-MAG3 scintigraphy shows very pale left kidney becoming increasingly better visualized later. Renographic curve of the left lidney shows obstruction over the third phase of the renogram.

**Figure 3:**
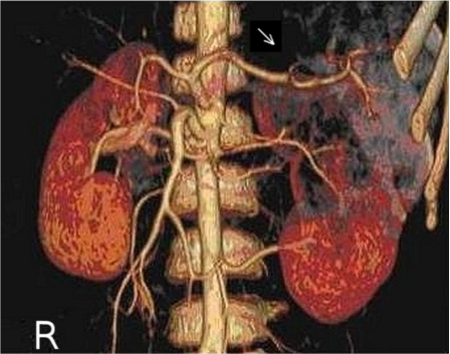
**Multi-slice computer tomography (MSCT) renal angiography of both kidneys in anteroposterior position shows severe left kidney damage (arrow) with 3 independent unobstructed arteries; two of them starting regularly, the third beginning caudally at the approximate position of the lower pole of the left kidney.** (R = right kidney).

A seven-year-old Croatian boy with hemophilia B with high-titer inhibitors to coagulation FIX was admitted at our institution with severe hematuria. The parents denied trauma, any medication or infection. He was previously treated with rFVIIa, mostly for bleeding affecting limb joints. Clinical, diagnostic and medication follow-up is shown in Figure 1. Painless hematuria was treated during the first three days with only symptomatic therapy consisting of intravenous hyperhidration and bed rest. On the fourth and fifth day, a fall in hemoglobin level was noticed and single daily dose of 285 μg/kg rFVIIa was administered intravenously in a 10–20 minute interval on both consecutive days. Despite the therapy, a life-threatening condition developed on the sixth day with rapid fall of red blood cells count (RBC) accompanied with massive hematuria. The total rFVIIa dose was subsequently increased by administration every three hours, four times in total with each amount of 105 μg/kg. The treatment successfully stabilized RBC count and reduced hematuria. As hematuria, although reduced, continued, for the following two days the child received additional rFVIIa (once daily 285 μg/kg). On the fourth day of rFVIIa therapy the patient first time complained of left lumbar colic pain, and visible blood clots in urine appeared. The rFVIIa therapy was discontinued. Only hyperhydration and occasional spasmolytic therapy were continued. From the eleventh day, hematuria was only microscopic. In the course of the disease several ultrasound (US) examinations were performed. Initially, normal US showed, coincidently with renal colics, enlarged left kidney with hyperechogenic inhomogenous parenchyma with partial loss of corticomedulary differentiation and dilated pelvicaliceal system with hyperechogenic inhomogenous content compatible with clots. Only a vascular bed over the left kidney without visualization of the parenchyma with practically afunctional renographic curve was found on ^99m^Tc-DTPA (Diethylene Triamine Pentacaetic Acid) renal scintigraphy (Figure 2A). In the first minutes of ^99m^Tc-MAG3 (Mercaptoacetyltriglycine) scintigraphy, the left kidney was very pale becoming increasingly better visualized later (Figure 2B). Renographic curve showed obstruction over the third phase of the renogram. MSCT (multi-slice computer tomography) renal angiography revealed severe left kidney damage with 3 independent unobstructed arteries; two of them starting regularly, the third beginning caudally at the approximate position of the lower pole of the left kidney. The same kidney had 2 veins who communicated with each other, the first had circumaortal course with vascular convolutes and the second (accessory) showed retroaortal course supplying the lower pole of the kidney (Figure 3). Nine months later renal scintigraphy was repeated. The finding was normal.

## Conclusion

The risk of clot formation is low even if rFVIIa was given in higher doses (>300 mcg/kg BW) [5,6]. But the treatment with high doses of rFVIIa on an off-label basis significantly increases the risk of arterial but not venous thromboembolic events among the elderly [7]. In the literature we found a report of a woman with Glanzmann Thrombasthenia with haematuria and clotting in renal pelvis after rFVIIa administration. In this case, the major reason for rFVIIa administration was intraoperative bleeding, during laparotomy for pelvic inflammatory disease. It is well known that inflammatory process activate "kinin-kallikrein" system and that leads to increased clotting [8].

Our patient had multiple renal vascular anomalies. Multiple renal arteries are not uncommon. Various reports estimate its incidence up to 32%, usually on the right side with very rare occurrence of triple arteries supplying one kidney [9-12]. Anatomical variations of multiple renal veins are less common with incidence ranging between 0.8 and 6% [13]. Both anomalies occur far more commonly on the right side [9,13,14]. Anomalies occurring in both arteries and vein together are extremely rare. To our knowledge, this is the first report of combination of both multiple arterial and vein kidney anomalies of one kidney while the other kidney had normal circulatory system. As we did not exceed recommended rFVIIa dosage and as right kidney was spared, the contribution of such vascular anomaly to the thromboembolic incident of the left kidney is a reasonable assumption. According to radionuclide examinations the main site of lesion and clotting event was intrarenal vascular bed, primarily in glomerular region, while tubular region was less affected. Renal damage secondary to pelvicaliceal obstruction is less likely.

Such an extremely rare troboembolic event in patients with hemophilia B certainly do not compromise safe administration of rFVIIa. However, caution is necessary if hematuria requires administration of rFVIIa. US color doppler renal imaging before and after drug administration should be sufficient as an early warning.

## Consent

Written informed consent was obtained from the patient’s parents for publication of this Case report and any accompanying images. A copy of the written consent is available for review by the Editor of this journal.

## References

[1] Astermark J (2003). Treatment of the bleeding inhibitor patient. Semin Thromb Hemost.

[2] Abshire T, Kenet G (2004). Recombinant factor VIIa: review of efficacy, dosing regimens and safety in patients with congenital and acquired factor VIII or IX inhibitors. J Thromb Haemostasis.

[3] Abshire T, Kenet G. Safety update on the use of recombinant factor VIIa and the treatment of congenital and acquired deficiency of factor VIII or IX with inhibitors. Haemophilia 2008; **14**(5):898-902. 10.1111/j.1365-2516.2008.01829.x 18684126

[4] Chuansumrit A, Angchaisuksiri P, Sirachainan N (2010). Critical appraisal of the role of recombinant activated factor VII in the treatment of hemophilia patients with inhibitors. J Blood Med.

[5] Salaj P, Brabec P, Penka M, Salaj P, Brabec P, Penka M, Pohlreichova V, Smejkal P, Cetkovsky P, Dusek L, Hedner U (2009). Effect of rFVIIa dose and time to treatment on patients with haemophilia and inhibitors:analysis of HemoRec registry data from the Czech Republic. Haemophilia.

[6] Young G, Cooper DL, Gut RZ (2012). Dosing and effectivness of recombinant activated factor VII (rFVIIa) in congenital haemophilia with inhibitors by bleed type and location: the experience of the Haemophilia and Thrombosis Research Society (HTRS) Registry (2004–2008). Haemaophilia.

[7] Levi M, Levy JH, Andersen HF, Truloff D (2010). Safety of Recombinant Activated Factor VII in Randomised Clinical Trials. N Engl J Med.

[8] Robinson KL, Savoia H, Street AM (2000). Thrombotic complications in two patients receiving NovoSeven. Haemophilia.

[9] Harrison LH, Flye MW, Seigler HF. **Incidence of anatomical variants in renal vasculature in the presence of normal renal function**. *Ann Surg* 1978; **188**(1):83-9. 10.1097/00000658-197807000-00014 PMC1396656 352280

[10] Mir NS, ul Hassan A, Rangrez R, Hamid S, Sabia, Tabish SA, Iqbal, Suhalia, **Massarat, Rasool Z. Bilateral duplication of renal vessels: anatomical, medical and surgical perspective**. *Int J Health Sci (Qassim)* 2008; **2**:179–185. PMC3068735 21475502

[11] Das S (2008). Anomalous renal arteries and its clinical imp'lications. Bratisl Lek Listy.

[12] Satyapal KS, Haffjee AA, Singh B, Ramsaroop L, Robbs JV, Kalideen JM (2001). Additional renal arteries incidence and morphometry. Surg Radiol Anat.

[13] Chavan SK, Wabale NR, Daimi RS (2010). Unusual variation of the renal vessels-a case report. Pravara Med Rev.

[14] Fernandes RMP, Conte FHP, Favorito LA, Abidu-Figueiredo M, Babinski MA (2005). Triple right renal vein: an uncommon variation. Int J Morphol.

